# TET Upregulation Leads to 5-Hydroxymethylation Enrichment in Hepatoblastoma

**DOI:** 10.3389/fgene.2019.00553

**Published:** 2019-06-12

**Authors:** Maria Prates Rivas, Talita Ferreira Marques Aguiar, Gustavo Ribeiro Fernandes, Luiz Carlos Caires-Júnior, Ernesto Goulart, Kayque Alves Telles-Silva, Monica Cypriano, Silvia Regina Caminada de Toledo, Carla Rosenberg, Dirce Maria Carraro, Cecilia Maria Lima da Costa, Isabela Werneck da Cunha, Ana Cristina Victorino Krepischi

**Affiliations:** ^1^Human Genome and Stem-Cell Research Center, Department of Genetics and Evolutionary Biology, Institute of Biosciences, University of São Paulo, São Paulo, Brazil; ^2^International Center of Research, A. C. Camargo Cancer Center, São Paulo, Brazil; ^3^Department of Biochemistry, Institute of Chemistry, University of São Paulo, São Paulo, Brazil; ^4^Department of Pediatrics, Support Group for Children and Adolescents With Cancer (GRAACC), Federal University of São Paulo, São Paulo, Brazil; ^5^Department of Pediatric Oncology, A. C. Camargo Cancer Center, São Paulo, Brazil; ^6^Department of Pathology, Rede D’Or São Luiz, São Paulo, Brazil

**Keywords:** hepatoblastoma, epigenetics, hydroxymethylation, active demethylation, DNA hypomethylation

## Abstract

Hepatoblastoma is an embryonal liver tumor carrying few genetic alterations. We previously disclosed in hepatoblastomas a genome-wide methylation dysfunction, characterized by hypermethylation at specific CpG islands, in addition to a low-level hypomethylation pattern in non-repetitive intergenic sequences, in comparison to non-tumoral liver tissues, shedding light into a crucial role for epigenetic dysregulation in this type of cancer. To explore the underlying mechanisms possibly related to aberrant epigenetic modifications, we evaluated the expression profile of a set of genes engaged in the epigenetic machinery related to DNA methylation (*DNMT1*, *DNMT3A*, *DNMT3B*, *DNMT3L*, *UHRF1*, *TET1*, *TET2*, and *TET3*), as well as the 5-hydroxymethylcytosine (5hmC) global level. We observed in hepatoblastomas a general disrupted expression of these genes from the epigenetic machinery, mainly *UHRF1*, *TET1*, and *TET2* upregulation, in association with an enrichment of 5hmC content. Our findings support a model of active demethylation by *TETs* in hepatoblastoma, probably during early stages of liver development, which in combination with *UHRF1* overexpression would lead to DNA hypomethylation and an increase in overall 5hmC content. Furthermore, our data suggest that decreased 5hmC content might be associated with poor survival rate, highlighting a pivotal role of epigenetics in hepatoblastoma development and progression.

## Introduction

Pediatric tumors are inherently different from tumors that develop in adults (Maris and Denny, 2002) since they are supposedly derived from defects of cell differentiation and organogenesis (Anderson, 2006). Embryonal tumors are a special class of childhood cancers characterized by a very early-onset exhibiting histological features that resemble developmental stages of the origin organ (Maris and Denny, 2002).

Cancer development involves the accumulation of genetic mutations over an extended period of time (Hanahan and Weinberg, 2011); however, this model is not suitable for pediatric cancer, which develops in a short temporal window, usually carrying a low number of somatic mutations (Vogelstein et al., 2013; Gröbner et al., 2018). A more comprehensive background takes into account the dysregulation of epigenetic mechanisms (Sandoval and Esteller, 2012), considering the interplay between genetic and epigenetic factors as a key element for tumor development (Soo You and Jones, 2012). Among the epigenetic regulators, DNA methylation, which occurs through the addition of a methyl group to a cytosine (5mC) by DNA methyltransferase enzymes (DNMTs) (Jones, 1999), has been extensively investigated. In tumor cells, well-known DNA methylation disturbances include repression of tumor suppressor genes by promoter hypermethylation, and hypomethylation at repetitive sequences, leading to genomic instability (Łuczak and Jagodzinski, 2006; Rodrigues-Paredez and Esteller, 2011).

Hepatoblastoma is a rare liver embryonal tumor, and the most common form of liver tumor in children (Wiederkehr et al., 2013; López-Terrada et al., 2014), with an incidence of 1-2.4 per million children each year (Paran and La Quaglia, 2009). These tumors carry few genetic alterations, mainly gains of chromosomes 2, 8, 20, and chromosome 18 deletion (Tomlinson and Kappler, 2012; Ozen et al., 2013; Rodrigues et al., 2014), and recurrent *CTNNB1* activating mutations; low frequency mutations of other *Wnt* pathway genes, such as *AXIN1, AXIN2, AXIN3* and *APC*, have also been reported (Tomlinson and Kappler, 2012; Eichenmüller et al., 2014; Jia et al., 2014). In a recent work from our group (Maschietto et al., 2017), the evaluation of hepatoblastoma methylomes disclosed a genome-wide methylation dysfunction, characterized by hypermethylation at specific CpG islands, in addition to a low-level hypomethylation pattern in non-repetitive intergenic sequences, the later finding corroborated by others (Cui et al., 2016).

DNA hydroxymethylation is a kind of DNA modification (Münzel et al., 2011) originated by 5mC conversion to 5-hydroxymethylcytosine (5hmC), in a process catalyzed by proteins encoded by *TET* genes. The 5hmC is an intermediate residue of the active demethylation process and seems to be related to pluripotency maintenance in stem cells and transcriptional activation (Wu and Zhang, 2011; Ko et al., 2011).

To elucidate the underlying mechanisms resulting in DNA methylation loss in hepatoblastomas, we evaluated the expression profiles of the *DNMT* and *TET* genes as well as the global level of 5hmC in a hepatoblastomas cohort.

## Materials and Methods

### Samples

Nineteen hepatoblastoma samples (HBs) and eight paired non-tumoral liver tissue samples were obtained from hepatoblastoma patients submitted to surgical resection in two pediatric cancer institutions (A. C. Camargo Cancer Center and GRAACC, São Paulo, Brazil). This study was carried out in accordance with the recommendations of Research Ethics Committee - A.C. Camargo Cancer Center, Brazil (registration number 1987/14), with written informed consent from all parents/guardian of participants. Clinical features of this HB cohort are described in Supplementary Table 1. All patients received pre-surgery chemotherapy according to the SIOPEL protocol^1^ and COG protocol^2^.

### Methods

#### Gene Expression Analysis

Microfluidics-based electrophoresis (Bioanalyzer, Agilent Technologies; CA, United States) was performed to verify quality, and only RNA samples with RNA Integrity Number (RIN) > 7.0 were used for gene expression analysis. The cDNA was synthesized with the High Capacity RNA-to-cDNA kit (Applied Biosystems, EUA) according to standard procedures. The experiment was designed and interpreted according to recommendations contained in International Guideline MIQE (The Minimum Information for Publication of Quantitative Real-Time PCR Experiments). The suitable housekeeping gene was chosen using the geNorm algorithm (Vandesompele et al., 2002) after expression analysis of *ACTB*, *GAPDH*, *B2M* genes and 18S ribosomal RNA (18S rRNA). Data were normalized using the expression values of the housekeeping gene 18S rRNA, and all reactions were performed with three technical replicates. The delta-delta *C*_t_ (ΔΔ*C*_t_) method was used for data analysis (Livak and Schmittgen, 2001). The Kruskal Wallis *post hoc* Dunn test with Bonferroni correction was used for statistical analysis in the GraphPad Prism 7 software.

### 5-Hydroxymethylcytosine (5hmC) Quantification

Nanodrop (Thermo Fisher Scientific, United States) was used to access quantity and integrity of DNA samples. The 5hmC global level quantification of 18 HBs and eigth paired non-tumoral liver tissue samples was carried out using the MethylFlash Hydroxymethylated DNA 5-hmC Quantification Kit (Epigentek, United States). The values of each sample are given as percentages relative to internal controls of the kit, according to the manufacturer’s recommendation. The *t*-test was used for the statistical analysis.

## Results

**FIGURE 1:**
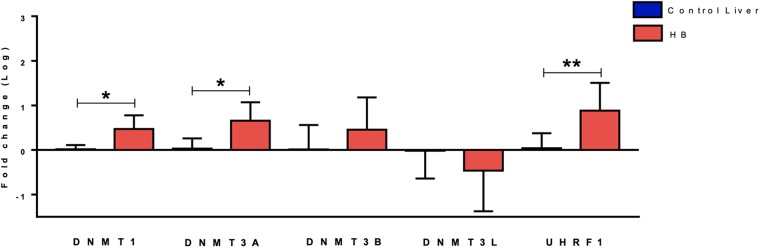
Expression analysis of DNA methylation genes in hepatoblastomas. *DNMT1, DNMT3A* and *UHRF1* upregulation in 19 tumor samples compared with eight non-tumoral liver samples as evidenced by RT-qPCR. Control Liver: non-tumoral liver tissue samples; HB: hepatoblastoma samples. Endogenous gene: *18S rRNA*. The Kruskal Wallis *post hoc* Dunn test with Bonferroni correction was used for statistical analysis. ^∗^*p*-value < 0.05, ^∗∗^*p*-value < 0.01.

Gene expression analysis of five DNA methylation genes (*DNMT1, DNMT3A, DNMT3B, DNMT3L*, and *UHRF1*) revealed up-regulation of *DNMT3A* (fold-change mean: 7.0; *p*-value: 0.024) and *DNMT1* (fold-change mean: 3.6; *p*-value: 0.012) expression in the group of 19 HBs as compared to eight paired non-tumoral liver tissues, while *DNMT3B* and *DNMT3L* expression values were not significantly altered (Figure 1). Data also showed a very significant increase in *UHRF1* expression in tumor samples (fold-change mean: 20.4; *p*-value: 0.015).

**FIGURE 2:**
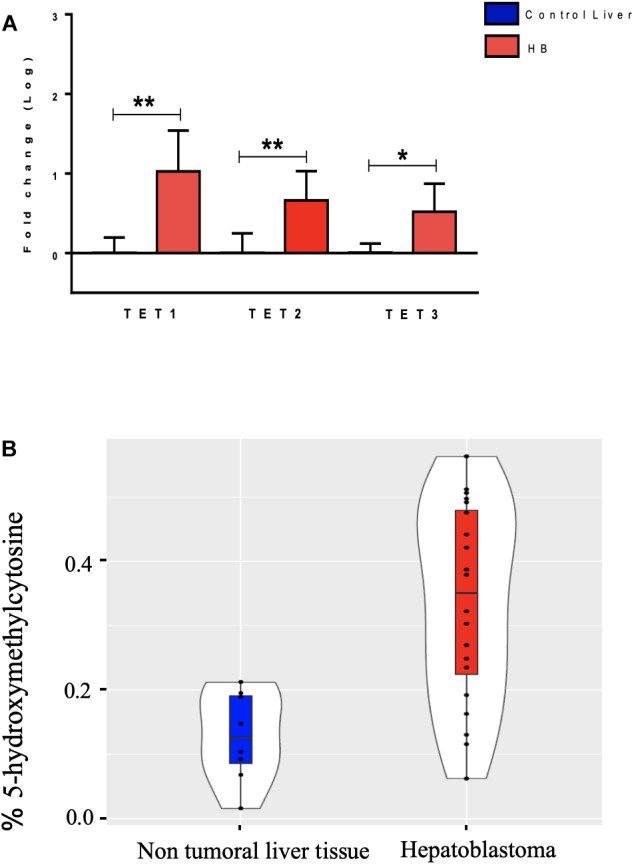
Analysis of DNA hydroxymethylation in hepatoblastomas. **(A)** Expression analysis of DNA hydroxymethylation genes in hepatoblastomas. Upregulation of three genes of the *TET* family in 19 tumor samples compared with eight non-tumoral liver samples as evidenced by RT-qPCR. Control Liver: non-tumoral liver tissue samples; HB: hepatoblastoma samples. Endogenous gene: *18S rRNA*. The Kruskal Wallis *post hoc* Dunn test with Bonferroni correction was used for statistical analysis. ^∗^*p*-value < 0.05; ^∗∗^*p*-value < 0.01. **(B)** 5hmC global level enrichment in hepatoblastoma samples. Violin plots showing the overall level of 5hmC in percentage in hepatoblastomas samples (red) and non-tumoral liver tissues (blue). Hepatoblastoma samples mean: 0.33%, SD: 0.15; non-tumoral liver tissue samples mean: 0.13%; SD: 0.07. The *t*-test was used for the statistical analysis. *p*-value: 4.2e-05.

**FIGURE 3:**
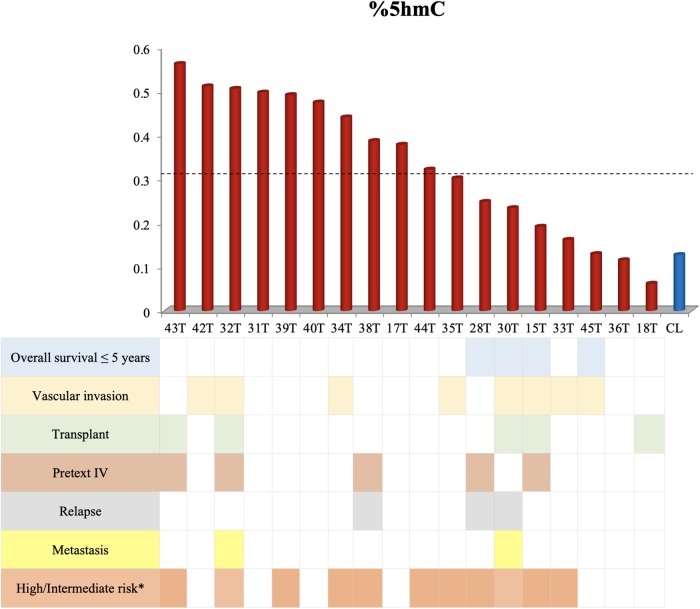
Decreased overall survival is associated with lower levels of 5hmC. This figure shows the 5hmC global content of 18 tumor samples in the columns and their respective clinical data below. Tumor samples with overall survival ≤ 5 years present reduced 5hmC content, below the mean of the tumor group (0.33% - represented by the dashed line) and close to the level of non-tumoral hepatic tissues (CL). The Fisher’s test was used for the statistical analysis. *p*-value 0.02. CL: non-tumoral liver tissue samples; T: hepatoblastoma tumor samples. ^∗^According to the CHIC criteria (Meyers et al., 2017).

The hydroxymethylation process was evaluated by assessing the expression level of the three *TET* genes, and all of them were found to be significantly upregulated in hepatoblastomas as compared to non-tumoral liver tissue (Figure 2A): *TET1* (fold-change mean: 21.5; *p*-value: 0.009), *TET2* (fold-change mean: 7.15; *p*-value: 0.009), and *TET3* (fold-change mean: 4.3; *p*-value: 0.024). Given the observed *TET* genes upregulation in HBs, the global content of 5hmC was evaluated in tumors and control hepatic tissues. Regardless of the variability in total 5hmC content seen across HBs, an overall enrichment of approximately 2.5× (*p*-value 4.2e-05; *t*-test) was observed in tumors (mean: 0.33%; SD: 0.15) relative to non-tumoral livers (mean: 0.13%; SD: 0.07) (Figure 2B). Statistical analysis based on the clinical data showed that patients with overall survival ≤ 5 years exhibited a 5hmC content lower than the mean of the tumor group (*p*-value 0.02; Fisher’s test) (Figure 3), closer to the level that was detected in the group of non-tumoral liver tissues.

## Discussion

We previously reported changes in the methylation profile of hepatoblastomas, with global hypomethylation in intergenic non-repetitive sequences, as well as hypermethylation at CpG islands, affecting genes associated with tumor suppression, lipid metabolism, and liver differentiation (Maschietto et al., 2017). Except for the *UHRF1* gene (Beck et al., 2018), here we disclosed for the first time dysregulated expression of genes from the DNA methylation machinery that could be related to the detected epigenetic disturbance, such as upregulation of *DNMT3A, DNMT1, UHRF1*, and the *TET* family of genes.

The interaction between UHRF1/DNMT1 plays a pivotal role in the inheritance of DNA methylation patterns, even though the details of this complex network remain under analysis. Thus, *UHRF1* is considered an essential epigenetic regulator for DNA methylation maintenance (Bostick et al., 2007). It is known that UHRF1/DNMT1 are involved in the maintenance of the hypermethylation of tumor suppressor gene promoters in cancer (Alhosin et al., 2016; Ashraf et al., 2017); in particular, overexpression of *UHRF1* was recently reported to silence specific tumor suppressor genes in hepatoblastomas (Beck et al., 2018). Indeed, enhanced *UHRF1* expression was identified in all cancers so far examined (Ashraf et al., 2017). Therefore, it is possible that *DNMT1* and *DNMT3A* upregulation observed in the present study contributes to the hypermethylation at specific CpG islands we previously reported in hepatoblastomas. Nevertheless, it has also been suggested that impaired interaction between UHRF1 and DNMT1 could be the origin of the global hypomethylation in cancer cells (Hervouet et al., 2010; Pacaud et al., 2014). Moreover, a different line of evidence revealed that *UHRF1* overexpression also acts as a negative regulator by degradation of the *de novo* DNA methylation protein, DNMT3A, as an additional mechanism leading to widespread DNA hypomethylation in cancer cells (Jia et al., 2016). Complementary to these findings, Mudbhary et al. (2014) demonstrated in both zebrafish hepatocytes and hepatocellular carcinoma that *UHRF1* overexpression promotes DNMT1 destabilization and de-localization, driving DNA hypomethylation; further, *UHRF1* increased expression was already associated with poor prognosis in hepatocellular carcinoma, contributing to cell proliferation and metastasis (Liu et al., 2017). These data further support the hypothesis that disruption of normal cooperation of DNA methylases and *UHRF1* can play a dual role in the patterns of DNA methylation in cancer cells, which mechanisms remain partially elusive. In combination with our results, these evidences suggest that, at least in liver cancer, including hepatoblastomas, the disclosed *UHRF1* overexpression may result in inefficient or impaired DNA methylation process, irrespective of the *DNMTs* expression level, contributing to the global low level hypomethylation pattern in non-repetitive intergenic sequences reported in hepatoblastomas (Cui et al., 2016; Maschietto et al., 2017) and hepatocellular carcinoma (Shen et al., 2013).

The conversion of methylated cytosine to 5hmC, mediated by *TET* enzymes, is the first step in the DNA demethylation active pathway (Wu and Zhang, 2011; Ko et al., 2011; Rasmussen and Helin, 2016). In adult cancer, a decrease in *TET* expression was reported to reduce the 5hmC level in solid and hematological tumors (Lian et al., 2012; Liu et al., 2013; Rawłuszko-Wieczorek et al., 2015; Rasmussen and Helin, 2016). However, the activity of these enzymes in pediatric tumors is poorly explored. Despite the limited size of our cohort, here we showed an increased expression of *TET* genes in hepatoblastomas, suggesting the occurrence of an active demethylation mechanism. Studies with animal models described *TET* overexpression in embryonic development stages repressing differentiation (Ito et al., 2010; Rawłuszko-Wieczorek et al., 2015; Rasmussen and Helin, 2016), as well as in cellular reprogramming of IPS cells (Bagci and Fisher, 2013). Thus, we can speculate that *TET* upregulation in hepatoblastomas indicates the occurrence of a blockage of liver differentiation, which corroborates the long-standing hypothesis of repression of the differentiation pathway of the origin organ leading to embryonal tumors genesis (Maris and Denny, 2002). A functional role for the observed *TET* overexpression was substantiated by the enrichment of the 5hmC content in these tumors, then partially explaining the DNA hypomethylation described in hepatoblastoma, in addition to the possible impaired *de novo* methylation caused by the observed *UHRF1* overexpression. An additional analysis regarding the underlying nature of the DNA sequences with 5hmC enrichment in hepatoblastomas (if intergenic non-repetitive or CpG islands) would add valuable information, and should be made in the future. Remarkably, in hepatocellular carcinoma, a reduced 5hmC content was directly associated with lower overall survival and poor prognosis (Liu et al., 2013). Our findings also suggested that lower levels of 5hmC in hepatoblastomas could be associated with decreased overall survival, highlighting the 5hmC content as a potential biomarker of risk for these tumors. However, these preliminary results require further validation in a larger cohort of hepatoblastomas.

This study addressed possible mechanisms leading to the hypomethylation pattern displayed by hepatoblastomas. Our data revealed disturbance in the expression of genes related to the DNA methylation machinery and enrichment of the 5hmC content in hepatoblastomas. As hepatoblastomas exhibit the lowest mutation rate reported so far for pediatric solid tumors (Gröbner et al., 2018), this analysis of genes of the epigenetic machinery provides evidence that epigenetic disruption is probably key for the onset of this liver embryonal tumor, and highlights the 5hmC content as a potential biomarker for poor overall survival.

## Data Availability

All datasets generated for this study are included in the manuscript and/or the Supplementary Files.

## Ethics Statement

This study was carried out in accordance with the recommendations of Research Ethics Committee – A.C. Camargo Cancer Center, Brazil, registration number 1987/14. All subjects gave written informed consent in accordance with the Declaration of Helsinki. The protocol was approved and all the samples were collected and extracted in the A.C.Camargo Cancer Center Bank of Macromolecules, following the technical and ethical procedures of the institution (Campos et al., 2012; Olivieri et al., 2014).

## Author Contributions

MR and AK conceived the study and participated in its design. MR, LC-J, EG, and KT-S performed the experiments and analyzed the data. GF performed statistical analysis. TA, CdC, IdC, MC, SdT, and DC evaluated patients, collected biological samples, and revised clinical data. MR and AK wrote the manuscript. CR and AK critically revised the manuscript. All authors have read and approved the final version of the manuscript.

## Conflict of Interest Statement

The authors declare that the research was conducted in the absence of any commercial or financial relationships that could be construed as a potential conflict of interest.

## Footnotes

1 http://www.siopel.org/

2 https://www.childrensoncologygroup.org/
